# Mechanical induction of cough in Idiopathic Pulmonary Fibrosis

**DOI:** 10.1186/1745-9974-7-2

**Published:** 2011-04-10

**Authors:** Richard M Jones, Simon Hilldrup, Benjamin DM Hope-Gill, Ronald Eccles, Nicholas K Harrison

**Affiliations:** 1 Respiratory Unit, Morriston Hospital, Heol Maes Eglwys, Swansea, SA6 6NL, UK; 2 Department of Respiratory Medicine, Llandough Hospital, Penlan Road, Cardiff, CF64 2XX, UK; 3 Common Cold Centre, Cardiff School of Biosciences, Cardiff University, Cardiff, CF10 3AX, UK; 4 School of Medicine, Swansea University, Swansea, SA2 8PP, UK; 5 Department of Respiratory Medicine, Nevill Hall Hospital, Brecon Road, Abergavenny, NP7 7EG, UK

## Abstract

**Background:**

Patients with idiopathic pulmonary fibrosis (IPF) frequently develop a dry, irritating cough which often proves refractory to anti-tussive therapies. The precise pathogenetic mechanisms responsible for this cough are unknown. We hypothesised that changes in nerves modulating mechanical sensitivity in areas of interstitial fibrosis might lead to enhanced cough response to mechanical stimulation of the chest in IPF.

**Methods:**

We studied 27 non-smoking subjects with IPF (63% male), mean (SD) age 71.7 (7) years and 30 healthy non-smokers. Quality of life (Leicester Cough Questionnaire), cough symptom scores and cough severity scores (visual analog scales) were recorded. Percussion stimulation was applied over the posterior lung base, upper anterior chest and manubrium sternum at sequential frequencies (20 Hertz (Hz), 40 Hz and 60 Hz) for up to 60 seconds and repeated twice at two minute intervals. The number of subjects achieving two and five-cough responses, total cough counts and cough latency were recorded. In separate experiments, the effect of mechanical stimulation on the pattern of breathing was determined in eight IPF subjects and five control subjects.

**Results:**

In patients with IPF, we demonstrated strong correlations between subjective cough measurements, particularly the cough symptom score and Leicester Cough Questionnaire (r = -0.86; p < 0.001). Mechanical percussion induced a true cough reflex in 23/27 (85%) IPF subjects, but only 5/30 (17%) controls (p < 0.001). More patients with IPF reached the two-cough response at a lower frequency (20 Hz) posteriorly than at other positions. Highest mean cough totals were seen with stimulation at or above 40 Hz. Mechanical stimulation had no effect on respiratory rate but increased tidal volume in four (50%) subjects with IPF, particularly at higher frequencies. It was associated with increased urge to cough followed by a true cough reflex.

**Conclusions:**

This study demonstrates that patients with IPF show enhanced cough reflex sensitivity to mechanical stimulation of the chest wall whilst normal individuals show little or no response. The observation that low frequency stimulation over the lung base, where fibrosis is most extensive, induces cough in more patients than at other sites supports the hypothesis that lung distortion contributes to the pathogenesis of cough in IPF.

## Background

Idiopathic pulmonary fibrosis (IPF) is a disease characterised by lung parenchymal distortion by fibroblastic proliferation with extracellular matrix deposition and an inflammatory cell infiltration. Patients typically present with progressive breathlessness but the majority develop an irritating cough during the course of the disease[1,2]. This cough is typically dry and proves resistant to conventional anti-tussive therapies[2].

The majority of respiratory diseases associated with cough, such as chronic bronchitis, asthma and acute viral infections, predominantly affect the airways or upper respiratory tract where sensory innervation is dense. By contrast, pathological changes in IPF principally affect the lung parenchyma and alveoli, where innervation is sparse. It is therefore surprising that cough is so common in this disorder. The mechanisms which cause cough in IPF are unknown but several theories have been proposed[3]. These include modulation of nerves in larger airways by neurotrophins generated within diseased lung parenchyma, mechanical lung distortion from fibrosis altering the activation of cough receptors and gastro-oesophageal reflux disease (GORD), which is known to be present in approximately 80% of patients with IPF[4].

Cough reflex sensitivity to chemical stimulation from inhaled capsaicin and substance P has been shown to be increased in patients with IPF, suggesting functional upregulation of pulmonary c-fibres[5,6]. However, as far as we are aware, there have been no studies of the cough response to mechanical stimulation of the lungs in IPF.

Crystal et al. [2] reported that 80% of surgical lung biopsies showing characteristic changes of usual interstitial pneumonia (UIP) had evidence of peribronchiolar fibrosis and/or inflammation, with the majority of biopsies displaying evidence of both narrowed and dilated airways. It is therefore possible that mechanical distortion of peripheral airway architecture could sensitise rapidly adapting receptors (RARs) in small airways thereby lowering the cough threshold. Alternatively, c-fibres in the pulmonary interstitium, which have been reported to inhibit the cough reflex in certain species, could be destroyed by the progressive fibrotic process[7,8].

Mechanical stimulation of the throat and trachea has been shown to induce cough in patients with upper respiratory tract infection but little or no cough in healthy subjects[9,10]. In one such study, chest wall vibration over the manubrium sternum was performed using a chest percussor originally developed to assist clearance of bronchial secretions[10]. This novel technique is potentially a non-invasive and safe method for inducing mechanical vibration of the underlying lung and hence physical deformation of sensory receptors such as RARs, independent of chemical stimuli.

In this context, the present study was devised with the following aims:

• To examine whether mechanical stimulation of the chest wall can induce cough in patients with IPF and if so, whether this response is reliable and reproducible.

• To assess whether varying the frequency of vibration and the site of stimulation induces different patterns of cough in patients with IPF.

• To correlate measures of any cough induced by mechanical stimulation with subjective measures of cough assessed by validated questionnaires.

• To determine whether mechanical stimulation of the chest wall has any effects on the pattern of breathing in patients with IPF or controls.

## Methods

### Patients

Twenty seven patients fulfilling the American Thoracic Society/European Respiratory Society criteria for the diagnosis of IPF were recruited[11]. All patients had clinical, radiological and physiological features consistent with a diagnosis of IPF. In addition, each patient had undergone high resolution computed tomography (HRCT) scanning confirming the presence of pulmonary fibrosis, with predominant reticular or honeycomb pattern in the subpleural regions of the lung bases and little or no ground glass shadowing. Five patients had also undergone a surgical lung biopsy which showed the histological pattern of UIP. All patients were either non-smokers or had not smoked for at least one year. Exclusion criteria are shown in table 1. Thirty healthy volunteers acted as control subjects. No patients or control subjects with a history of gastro-oesophageal reflux were included unless they were established on proton pump inhibitor therapy for at least one month prior to the study commencement. The Leeds Dyspepsia Questionnaire (LDQ), an eight item symptom-based questionnaire was used to assess the severity of dyspepsia[12]. The possible total score ranges from 0 - 40, with lower values indicating less and higher values more severe dyspepsia. Measurement of pulmonary function was performed according to published guidelines[13]. Ethical approval was granted by the Local Research Ethics Committee prior to the study commencing (South West Wales Research Ethics Committee, reference number 07/WMW02/111) and informed written consent was provided by all subjects. The research was carried out in accordance with the Helsinki Declaration.

**Table 1:** Study Exclusion Criteria

**1**	History of smoking within 1 year
**2**	Evidence of respiratory tract infection within 6 weeks
**3**	History of untreated rhino-sinusitis
**4**	Untreated gastro-oesophageal reflux disease
**5**	Asthma or other respiratory disease other than IPF
**6**	History of asbestos exposure
**7**	History of collagen vascular diseases
**8**	Other severe, systemic co-morbidity
**9**	Drug therapy with angiotensin-converting enzyme inhibitors
**10**	Chest wall deformity precluding mechanical percussion

### Subjective Assessment of Cough

Three separate measures of cough were undertaken. All subjects were asked to grade their cough severity from 0 (no cough) to 100 (worst cough ever) using a 100 mm linear visual analogue scale (VAS)[14]. The impact of cough on quality of life was assessed using the Leicester Cough Questionnaire (LCQ)[15]. This consists of 19 questions, divided into three domains (social, psychological and physical) with a total calculated score ranging from 3-21, with higher scores indicating less impact on quality of life. Severity of daytime and night cough symptoms were also assessed using the cough symptom score (CSS) (table 2)[16]. Daytime and night time scores were added with a total possible score of ten.

**Table 2:** Cough symptom score

Score	Day	Night
**0**	No cough	No cough
**1**	Cough for one short period	Cough on waking only
**2**	Cough for two or more short periods	Wake once or early due to cough
**3**	Frequent coughing, which did not interfere with usual daytime activities	Frequent waking due to cough
**4**	Frequent coughing, interfering with usual daytime activities	Frequent coughs most of the night
**5**	Distressing cough for most of the day	Distressing cough most of the night

### Mechanical cough stimulation

**Figure 1:**
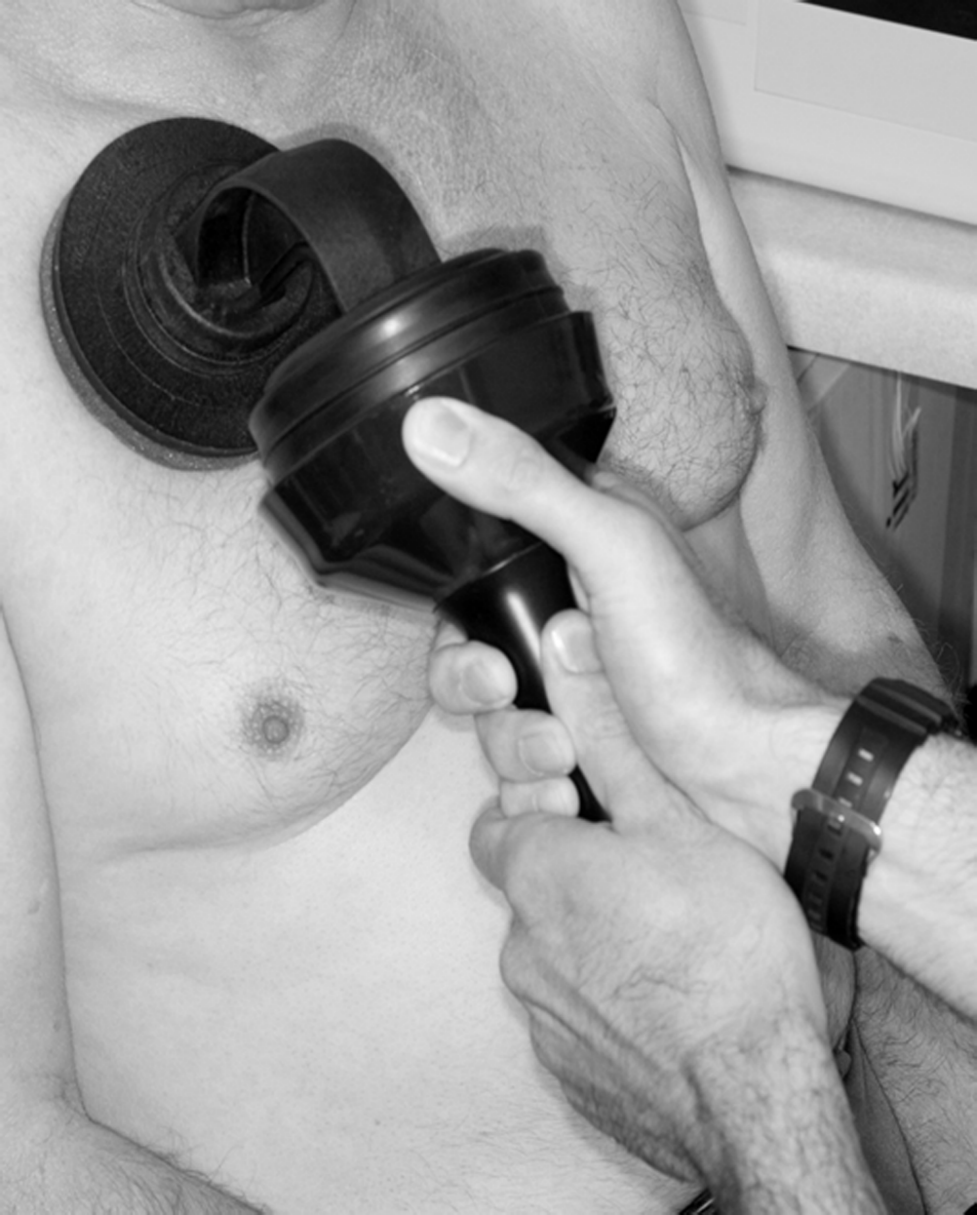
**Study subject undergoing mechanical percussion of the chest using a G5 Variko percussor (Physiotherapie Generale, Casteljaloux, France)**.

Subjects underwent mechanical stimulation of the chest wall using a percussor (G5 Variko; Physiotherapie Generale, Casteljaloux, France). A soft sponge rubber applicator (applicator no. 212/diameter 100 mm and thickness 30 mm; Physiotherapie Generale, Casteljaloux, France) was used to apply the percussor to the chest wall. Use of a right angle directional stroking adaptor converted the oscillatory effect produced by the face plate to an optimal percussive effect with stimulation frequencies from 20 to 60 Hz (Figure 1). A microphone connected to a computer recorded sound signals. Manual cough counting was conducted using a digital audio editing package with an audiovisual display (Audacity 1.2.6 software, Pittsburgh, USA). Coughs were identified as characteristic explosive sounds[17].

Subjects attended the laboratory between 09:00 and 11:00, following abstinence from caffeine-containing drinks for at least six hours and fasted for at least two hours. They were asked to sit comfortably on a chair for a six minute acclimatisation period during which their spontaneous cough frequency was measured and subsequently used to calculate the background cough frequency. Each subject was asked to rotate 90 degrees in the chair and the percussor was applied with a uniform pressure, sequentially to the following areas of the chest wall:

• the base of the right lung in the posterior axillary line,

• the anterior right chest over the 2^nd ^intercostal space in the mid-clavicular line and

• over the manubrium sternum.

Using an initial stimulation frequency of 20 Hz, percussion was applied for a maximum of one minute but switched off if the subject coughed within the one minute period. Any vibration-induced cough that occurred within two minutes from the start of percussion was counted. After two minutes, this procedure was then repeated and at each area of the chest wall in triplicate. The total number of coughs in the three stimulation periods (corrected for background cough), was recorded as the six minute cough frequency. The number of subjects who achieved a two-cough and a five-cough response were recorded as C2 and C5 cough thresholds respectively. For determination of C2 and C5 responses, only coughs occurring during or within 15 seconds of cessation of mechanical stimulation were counted, in accordance with guidelines for other cough challenges[17]. Cough latency (time to first cough) was also recorded. Percussion was then repeated in an identical manner at stimulation frequencies of 40 Hz and 60 Hz in immediate succession. Subjects who did not cough were recorded as non-responders. To assess the reproducibility of the technique, six patients with IPF underwent a repeat study using the above protocol at least one week after the initial study.

To determine any effects of mechanical stimulation on the pattern of breathing, the above protocol was repeated in eight patients with IPF (six who had previously coughed in response to mechanical stimulation and two who had not) and five normal volunteers using a portable recording device designed for the diagnostic assessment of cardio-respiratory sleep disorders (Alice PDx Diagnostic Systems, Philips-Respironics, Murrysville, PA, USA). This equipment provides measurement of: airflow from a nasal pressure cannula and oral thermistor; movements of abdominal and chest wall from effort belts, oxygen saturation and pulse using pulse oximetry. A built-in microphone also records associated sounds simultaneously. At the end of the recording period, all subjects were asked to describe subjective feelings of urge to cough, change in pattern of breathing or feelings of breathlessness.

### Statistical analysis

Total cough counts during six minutes of stimulation are expressed as mean (SEM) and compared by the unpaired t-test. The unpaired *t *test was also used to compare baseline variables between groups. Non-parametric data are expressed as median (IQR) and compared by the Mann-Whitney U test and Wilcoxon's signed rank test. When analysing multiple comparisons of mean cough counts at different stimulation frequencies within individuals, a repeated measures one-way analysis of variance (ANOVA) was applied to normally distributed data with Tukey's pairwise analysis for determining true differences. Fisher's exact test was used to analyse categorical data and Spearman's rank correlation coefficient to assess association between variables[18]. p values less than 0.05 were considered statistically significant[18]. All data was analysed by using GraphPad Prism 5 (GraphPad Software Inc., CA, USA).

## Results

### Subject characteristics

Baseline demographic and pulmonary function data for patients and control subjects is shown in table 3. There was no significant difference in symptoms of gastro-oesophageal reflux between patients and controls subjects although more patients with IPF were taking anti-reflux medication (67%) compared to control subjects (23%), p = 0.001.

**Table 3:** Baseline characteristics of the study subjects

	Control subjects	IPF subjects	
	n = 30	n = 27	p Value
Age, years	65.6 (5.3)	71.7 (7)	<0.001
Sex, male : female	21 : 9	17 : 10	0.589
Body Mass Index, kg/m^2^	26.3 ± 3.5	29.3 ± 4.6	0.007
Ever smoking: (% with ≥1 pack-year)	37	59	0.114
FEV_1_, % predicted	108.2 (10.9)	79.1 (18.5)	<0.001
FVC, % predicted	120.6 (13.7)	80.4 (20.9)	<0.001
DL_CO _,% predicted	86.5 (11.9)	43.7 (12)	<0.001
TLC, % predicted	ND	61.9 (12)	
LDQ score, median (IQR)	0 (0-2)	2 (0-4)	0.06
Corticosteroid use, n (%)	0 (0)	9 (30)	<0.001

Data are presented as mean (SD) unless indicated otherwise

*Definition of abbreviations: *IPF = idiopathic pulmonary fibrosis; FEV_1 _= forced expiratory volume in 1 s; FVC = forced vital capacity; DL_CO _= lung diffusion capacity for carbon monoxide; TLC = total lung capacity; ND = not determined; VAS = visual analogue scale; IQR = interquartile range; LDQ = Leeds dyspepsia questionnaire.

### Subjective measures of cough

**Figure 2:**
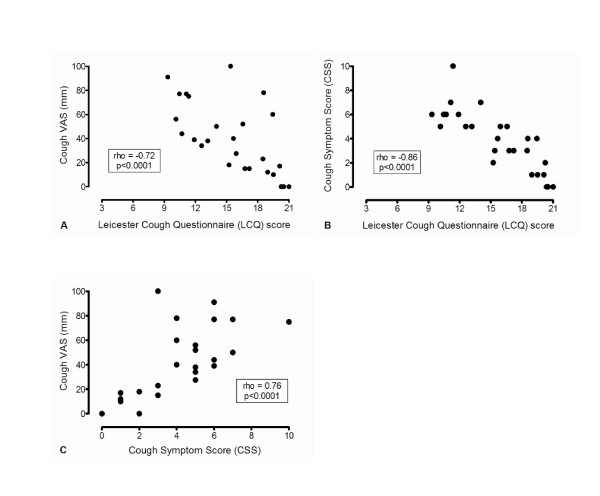
**Subjective cough assessment in patients with IPF**. a) Relationship between Leicester Cough Questionnaire and VAS scores. b) Relationship between Leicester Cough Questionnaire and cough symptom scores. c) Relationship between VAS and cough symptom scores.

Compared with controls, IPF patients had significantly greater median cough symptom scores than control subjects as assessed by VAS (38 [15-60] v 0 [0-4.5]; p < 0.001) and CSS (4 [2-6] v 0 [0-0]; p < 0.0001) and lower median LCQ scores (15.9 [11.9-19.5] v 20.8 [20.5-21]; p < 0.001). In patients with IPF, there were strong negative correlations between the LCQ score and both the CSS and VAS scores, with a further strong positive correlation between the VAS and CSS (Figure 2). There was no correlation between any subjective measures of cough and any measurement of pulmonary function.

### Cough induction with mechanical percussion

In healthy subjects, mechanical percussion induced very little cough and 25 out of 30 subjects (85%) exhibited no cough at any frequency at any site of stimulation. By comparison, 23 out of 27 patients (80%) with IPF coughed on percussive stimulation (p < 0.0001). Of the four patients with IPF who did not cough at all, three were receiving prednisolone.

### Cough Threshold

#### • Posterior chest

**Figure 3:**
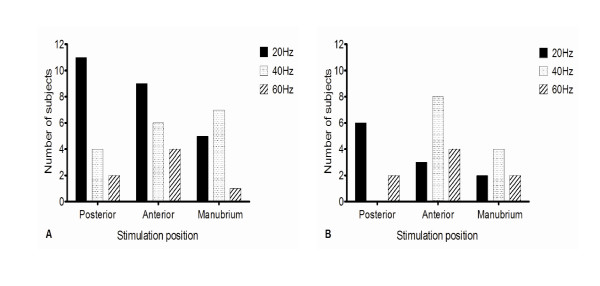
**Threshold frequencies at which**: a) two-cough responses. b) five-cough responses were induced in each stimulation position in patients with IPF.

Of the IPF patients, 17 out of 27 reached a two-cough response during an individual period of mechanical stimulation. This was detected in eleven subjects at 20 Hz, four at 40 Hz and two at 60 Hz (Figure 3a). A five-cough response was only achieved by eight subjects with IPF (Figure 3b). No healthy subjects reached a two or five-cough response in response to stimulation at this site at any frequency.

#### • Anterior chest

Nineteen subjects with IPF achieved a two-cough response. This was achieved by nine subjects at 20 Hz, six at 40 Hz and four at 60 Hz (Figure 3a). In fifteen of these subjects, a five-cough response was induced during at least one period of mechanical stimulation (Figure 3b). Four healthy subjects exhibited a two-cough response to mechanical stimulation, at 40 Hz in two individuals and 60 Hz in two others. None exhibited a five-cough response.

#### • Manubrium sternum

Of the IPF group, 13 subjects demonstrated a two-cough response. This occurred in five subjects at 20 Hz, seven at 40 Hz and one at 60 Hz (Figure 3a). In eight of these individuals, a five-cough response was also detected (Figure 3b). Only one healthy subject demonstrated both a two and five-cough response, which was in response to stimulation at 20 Hz.

### Total Cough Count

**Figure 4:**
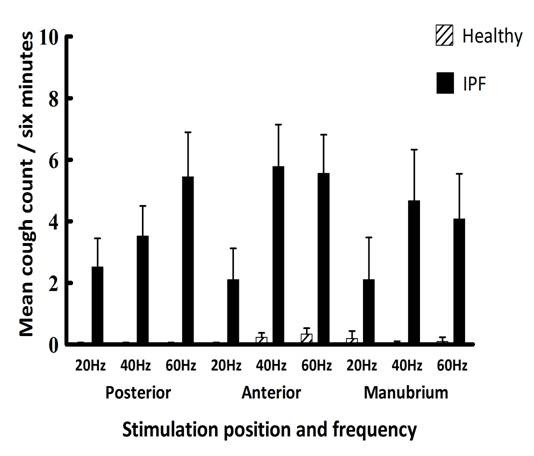
**Mean (±SEM) total cough count in patients with IPF and controls at different sites of stimulation on the chest wall and at different frequencies**.

The mean total cough counts (corrected for background cough) for the total three stimulation episodes at different sites of stimulation and different frequencies are shown in Figure 4. There was a significant difference between the mean cough counts of IPF patients and healthy subjects in each corresponding stimulation position and at all stimulation frequencies (p < 0.01). Applying stimulation of increasing frequencies to the posterior chest resulted in significant sequentially higher mean cough counts in IPF patients (p < 0.01). Significant differences were shown between the mean total cough counts at 20 Hz and 60 Hz (p < 0.01) and those at 40 Hz and 60 Hz (p < 0.05). Over the anterior chest and manubrium sternum, mean cough counts appeared greatest at stimulation frequencies of 40 and 60 Hz. There was no significant difference in mean total cough counts at different frequencies of stimulation over the manubrium sternum, but significant differences were seen between the mean total cough counts at 20 Hz and 40 Hz and between 20 Hz and 60 Hz stimulation over the anterior chest (p < 0.01).

### Cough Latency

In IPF subjects who coughed in response to mechanical stimulation, the mean time to first cough (cough latency) ranged from 32.7 to 57.3 seconds at each stimulation frequency in each location tested. No clear pattern emerged in relation to site of chest wall stimulated, frequency of stimulation or subjective measures of cough (data not shown).

### Relationship between total cough counts, subjective cough scores and lung function tests

The correlation between individual IPF patients' total cough counts at different sites and frequencies of percussion and the three subjective cough symptom scores are shown in table 4. There were particularly good correlations between total cough counts and the CSS. By contrast, there was no correlation between cough counts and any measure of lung function (FEV_1_, FVC, TLC or DL_CO_).

**Table 4:** Relationship between total six minute cough counts and subjective cough scores (LCQ, CSS and cough VAS) in subjects with IPF

	Total six minute cough count								
	
	Posterior Chest			Anterior chest			Manubrium sternum		
	
	20 Hz	40 Hz	60 Hz	20 Hz	40 Hz	60 Hz	20 Hz	40 Hz	60 Hz
**LCQ**	r = -0.29	r = -0.42	r = -0.24	r = -0.2	r = -0.42	r = -0.65	r = -0.06	r = -0.31	r = -0.41
	p = 0.14	p = 0.028*	p = 0.231	p = 0.324	p = 0.028*	p =< 0.001*	p = 0.758	p = 0.116	p = 0.032*

**CSS**	r = 0.4	r = 0.51	r = 0.4	r = 0.39	r = 0.62	r = 0.73	r = 0.09	r = 0.48	r = 0.49
	p = 0.038*	p = 0.007*	p = 0.038*	p = 0.043*	p =< 0.001*	p =< 0.001*	p = 0.638	p = 0.011*	p = 0.01*

**VAS**	r = 0.34	r = 0.28	r = 0.23	r = 0.22	r = 0.43	r = 0.63	r = 0.14	r = 0.23	r = 0.18
	p = 0.079	p = 0.158	p = 0.241	p = 0.28	p = 0.024*	p =< 0.001*	p = 0.49	p = 0.243	p = 0.373

*p values < 0.05

### Reproducibility of mechanically induced cough

**Figure 5:**
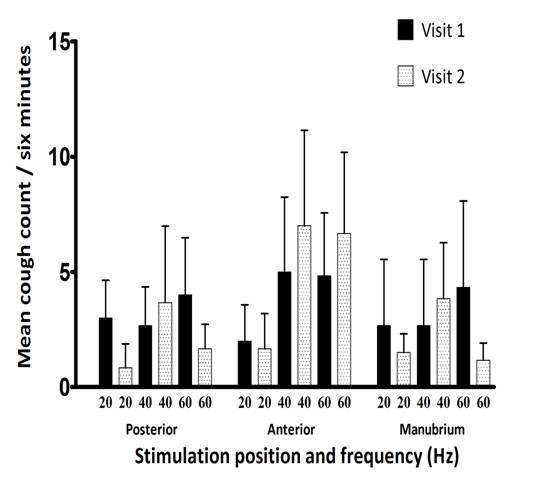
**Reproducibility of mean (±SEM) total cough counts in patients with IPF measured on two occasions one week apart**.

Repeat experiments under identical conditions in six patients with IPF studied at least one week apart suggested there was some variability in mean total cough counts and C2 cough threshold (Figure 5). However, there was no statistical difference in mean total cough counts at each location and frequency measured on the two occasions (p > 0.05), although the numbers analysed were small.

### Effects of mechanical stimulation on patterns of breathing

**Figure 6:**
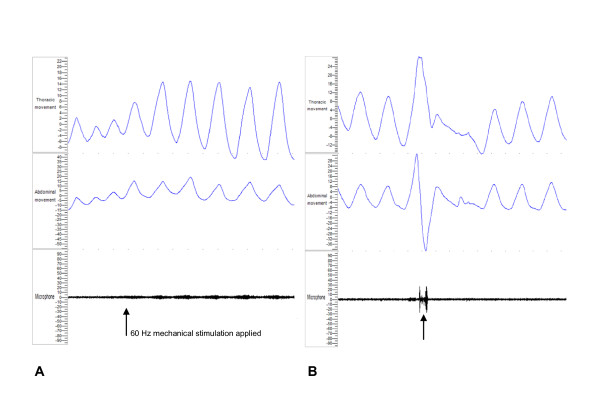
**Respiratory polygraphy recordings obtained from a subject with IPF demonstrating**: a) Increased tidal volume on thoracic and abdominal effort belt recordings following initiation (arrow) of 60 Hz mechanical stimulation to the posterior lung base. b) Increased inspiratory effort on thoracic movement sensors preceding a single true three-phase cough (arrow) during 40 Hz mechanical chest wall stimulation to the posterior lung base.

In healthy subjects, there was no change in rate or depth of respiration at any stimulation frequency. Similarly, in patients with IPF, no change in respiratory rate was observed. However, an increased tidal volume was clearly demonstrated in four patients (50%) in response to mechanical stimulation and this became more pronounced at higher frequencies (Figure 6a). It was associated with a subjective increase in urge to cough followed by a true cough reflex characterised by a preceding large inspiratory effort (Figure 6b). These four and two further patients commented on an increased urge to breathe more deeply with increasing mechanical stimulation in all areas. Interestingly, all six had shown a cough response whilst the two patients who did not report this sensation did not cough. One healthy subject spontaneously reported a mild urge to breathe more deeply with stimulation applied to the anterior chest at 40 and 60 Hz.

## Discussion

This is the first study to investigate the effects of mechanical chest wall percussion on the cough reflex and patterns of breathing in patients with IPF. The technique appears to induce a true cough rather than an expiratory reflex in the majority of subjects with IPF, but has little or no effect on healthy controls in whom any cough was minor and short-lived. The latter observation is consistent with previous studies in healthy humans[10,19,20]. In particular, the observation that low frequency stimulation (20 Hz) over the lung base (where fibrosis is usually most extensive in IPF) induces a C2 cough response in more patients than at other sites is consistent with the hypothesis that distortion of lung architecture contributes to the pathogenesis of cough in IPF, possibly by a mechanism involving rapidly adapting receptors (RARs).

RARs terminate in the intrapulmonary airways of all mammalian species and are exquisitely sensitive to mechanical stimulation[21-23]. They are dynamic, afferent receptors that demonstrate sustained rapid activation to alterations in airway mechanics, but are relatively insensitive to capsaicin or inflammatory mediators such as histamine, bradykinin and prostaglandins[24]. Studies on animals suggest that reduced lung compliance increases the discharge rate of RARs[25]. RARs demonstrate rapid adaptation (1-2 seconds) to continued lung inflation and are very responsive to changes in dynamic lung compliance[22]. It is therefore possible that reduced lung compliance in IPF may alter the firing rate of RARs resulting in an enhanced cough sensitivity as demonstrated in this study. Another possible explanation for our observations is that transmission of vibrated impulses from peripheral lung parenchyma to better innervated larger bronchi is enhanced in fibrotic lungs, thereby providing a greater mechanical stimulus to induce cough.

In this study, we excluded patients with chest wall deformities in order to minimise this as a confounding factor. However, stimulation over different areas of the chest wall may result in variability in the percussive stimulus exerted on the underlying lung due to differences in chest wall anatomy or thickness. As a group, the Body Mass Index of our patients with IPF was significantly greater than controls. Thus, any reduced transmission of the vibration due to increased chest wall thickness would favour a null hypothesis.

We cannot be certain that direct stimulation of RARs by mechanical vibration contributed to the cough response seen in patients with IPF. Indeed, the precise distribution and structure of RAR terminations in the intrapulmonary airways of patients with this condition are unknown [21,22]. Furthermore, the vibratory stimulus applied in these experiments would likely result in stimulation of sensory nerves in both chest wall and airways and it is plausible that chest wall receptors in IPF may be altered by changes in the lung mechanics and volumes seen in this disease. However, by excluding smokers, subjects with concurrent respiratory disease and other causes of cough, we attempted to minimise the possible confounding factor of mucus in the distal airways stimulating mechanical receptors, although this cannot be entirely ruled out. Finally, in preliminary studies on patients using the percussor without (sham vibration stimulus) and with the right angle directional stroking adaptor (true vibration stimulus) to optimise the oscillatory effect, cough was only induced when the adaptor was applied whilst in sham experiments, no cough was observed.

This implies the mechanical vibration applied in the subsequent experiments was a true stimulatory impulse.

In contrast to the C2 response, fewer patients with IPF achieved the C5 threshold at any frequency. These results are similar to those of a previous study which used chemical stimulation with capsaicin to induce cough in IPF patients[6]. This observation could be explained by the adaptation of RARs following their initial activation resulting in a short-lived cough response. Another possibility is that some individuals voluntarily suppress cough, an observation previously noted in normal volunteers inhaling capsaicin,[26] whilst others may cough in more prolonged paroxysms. These findings add further support to the notion that the two-cough response is a more reliable measure of cough reflex sensitivity in IPF than the C5 response[6].

Our measurements of chest and abdominal wall movement and airflow at the mouth together with audio-visual recordings demonstrated that the cough we induced by vibration was indeed a true cough and not an expiratory reflex. Interestingly, it appears to be preceded by an increase in tidal volume but not rate of respiration and then followed by an urge to cough. The explanation as to why this should occur is uncertain but may relate to variation in density or sensitivity of RARs between individuals. In a previous study on chest wall vibration in five patients with asthma, the subjects described a sensation of breathlessness similar to an acute exacerbation of asthma but no cough was reported [20]. To our knowledge, there are no reports describing this technique to study cough in patients with other respiratory conditions such as COPD, bronchiectasis or idiopathic cough.

It is noteworthy that a previous study reported an increased cough threshold to citric acid in healthy volunteers during simultaneous vibratory stimulation of the chest wall anteriorly in the right second intercostal space[27]. The authors postulated that this inhibition of cough occurred due to chest wall afferent nerves affecting higher centres. However, the stimulation frequency of 100 Hz used in that study was greater than the maximum frequency that could be achieved by the percussor used in the present study. It is possible that mechanical induction of cough depends upon the balance of inhibitory chest wall afferents and stimulatory RARs within the lung and that the latter predominated in our experiments using lower frequencies.

The total cough response (six minute cough count) in patients with IPF was greater when higher frequency stimulation (40 and 60 Hz) was applied to all three areas of the chest wall, whilst again normal controls showed little or no cough response. This may indicate there is a threshold frequency for induction of cough which is lowered in patients with IPF. Interestingly, the total cough count correlated with some subjective assessments of cough severity, particularly the CSS. A recent study demonstrated strong correlations between objective 24 hour cough frequency and both the VAS and LCQ scores in patients with IPF[28]. These findings suggest that in IPF, mechanical stimulation may be a good method for discerning cough of clinical relevance to patients' quality of life compared to chemical methods which induce cough in all subjects[5,6].

In the present study, patients were asked to provide three different subjective measure of cough severity, all of which have been previously validated. The median VAS score of 38 mm was similar to results from previous studies[5,6,28,29]. Our findings using the LCQ confirm that cough results in at least moderate impairment in quality of life for a significant number of patients with IPF. Furthermore, the LCQ correlated closely with the CSS. Indeed, all three subjective measure of cough showed good correlation. The CSS also indicated that patients cough significantly less at night than during the daytime, as recently confirmed in a study of diurnal objective cough counts in patients with IPF[28].

Previous studies in IPF have been unable to demonstrate a correlation between the VAS cough score and measures of disease severity assessed by pulmonary function[5,6]. The present study similarly failed to demonstrate a relationship between any subjective cough scores and measurements of pulmonary function. These findings would suggest that cough is not a good marker for the severity of IPF and highlight the likely heterogeneity of mechanisms causing cough in this condition, with possible confounders being GORD and corticosteroid therapy. It is possible that HRCT scanning may prove more useful in this regard. However, whilst all our patients underwent HRCT scanning for diagnostic purposes, contemporaneous scans were unavailable for the majority of patients and we did not feel that repeat imaging was justifiable merely for the purpose of correlation.

## Conclusion

In summary, this study demonstrates that patients with IPF show an enhanced cough reflex sensitivity to mechanical stimulation of the chest wall. It also shows there is a good correlation between cough induced by mechanical stimulation and subjective measures of cough severity, particularly the CSS. The observation that low frequency stimulation of the postero-basal lung base, where fibrosis is often most extensive, induces cough in more patients than at other sites is consistent with the hypothesis that lung distortion is a contributory factor to the pathogenesis of cough in IPF, possibly by activating RARs or destruction of inhibitory c-fibres. The use of chest wall percussion to induce a true cough reflex may prove a useful additional method for assessing novel anti-tussive therapies for cough in patients with IPF.

## Competing interests

The authors declare that they have no competing interests.

## Authors' contributions

RMJ contributed to the design of the study, data collection, analysis and interpretation and drafting/revising of the manuscript. SH was also involved in the data collection. RMJ, NKH and RE were involved in drafting and revising the manuscript. NKH, SH, BDMHG and RE were all involved in the study design and conception and provided substantial input into the analysis and interpretation of the data. All authors have read and approved the final version.
